# The Non-Catalytic Carboxyl-Terminal Domain of ARFGAP1 Regulates Actin Cytoskeleton Reorganization by Antagonizing the Activation of Rac1

**DOI:** 10.1371/journal.pone.0018458

**Published:** 2011-04-04

**Authors:** Ka Yu Siu, Mei Kuen Yu, Xinggang Wu, Min Zong, Michael G. Roth, Hsiao Chang Chan, Sidney Yu

**Affiliations:** 1 School of Biomedical Sciences, The Chinese University of Hong Kong, Shatin, New Territories, Hong Kong; 2 Epithelial Cell Biology Research Center, The Chinese University of Hong Kong, Shatin, New Territories, Hong Kong; 3 Department of Biochemistry, University of Texas Southwestern Medical Center at Dallas, Dallas, Texas, United States of America; University of Birmingham, United Kingdom

## Abstract

**Background:**

The regulation of the actin cytoskeleton and membrane trafficking is coordinated in mammalian cells. One of the regulators of membrane traffic, the small GTP-binding protein ARF1, also activates phosphatidylinositol kinases that in turn affect actin polymerization. ARFGAP1 is a GTPase activating protein (GAP) for ARF1 that is found on Golgi membranes. We present evidence that ARFGAP1 not only serves as a GAP for ARF1, but also can affect the actin cytoskeleton.

**Principal Findings:**

As cells attach to a culture dish foci of actin appear prior to the cells flattening and spreading. We have observed that overexpression of a truncated ARFGAP1 that lacks catalytic activity for ARF, called GAP273, caused these foci to persist for much longer periods than non-transfected cells. This phenomenon was dependent on the level of GAP273 expression. Furthermore, cell spreading after re-plating or cell migration into a previously scraped area was inhibited in cells transfected with GAP273. Live cell imaging of such cells revealed that actin-rich membrane blebs formed that seldom made protrusions of actin spikes or membrane ruffles, suggesting that GAP273 interfered with the regulation of actin dynamics during cell spreading. The over-expression of constitutively active alleles of ARF6 and Rac1 suppressed the effect of GAP273 on actin. In addition, the activation of Rac1 by serum, but not that of RhoA or ARF6, was inhibited in cells over-expressing GAP273, suggesting that Rac1 is a likely downstream effector of ARFGAP1. The carboxyl terminal 65 residues of ARFGAP1 were sufficient to produce the effects on actin and cell spreading in transfected cells and co-localized with cortical actin foci.

**Conclusions:**

ARFGAP1 functions as an inhibitor upstream of Rac1 in regulating actin cytoskeleton. In addition to its GAP catalytic domain and Golgi binding domain, it also has an actin regulation domain in the carboxyl-terminal portion of the protein.

## Introduction

The small GTPase ARF serves as a key regulator of a number of cellular processes including vesicle trafficking, signal transduction and regulation of the actin cytoskeleton [1]. The six isoforms of ARF in mammals can be categorized into three classes: Class I (ARF1, 2 and 3), Class II (ARF4 and 5) and Class III (ARF6). Among them, ARF1, the most extensively studied Class I member, regulates the formation of coated vesicles on secretory and endocytic membranes [2] and also take part in signal transduction in a number of signaling pathways [3], [4], [5], [6], [7], [8], [9], [10], [11], [12]. ARF6 functions in the endocytic pathway and regulates actin cytoskeleton [2]. Because of their diverse and complex cellular functions, the activity of ARF proteins is highly regulated. Like other small GTPases, ARFs cycle between an active, GTP-bound form and an inactive, GDP-bound form. The intrinsic GTPase activity of ARF is negligible [7] and the inactivation of ARF requires interactions with GTPase activating proteins (ARF GAPs). All ARF GAPs have an ARF GAP catalytic domain of 120 amino acids enriched in cysteine. To date, there are at least 24 such sequences identified in the human genome [9] and the genes containing them are divided into 10 subfamilies. With the exception of the ADAP subfamily, at least one member within all other subfamilies have been demonstrated experimentally to have ARF GAP activity [13]. Other than the ARF GAP catalytic domain, these genes are vastly different in terms of their size, the identifiable features that they contain and their subcellular localization [9]. The existence of multiple domains in these molecules not only reflects the complex functions of ARF but also indicates that these ARF-GAPs have functions in addition to stimulating GTP hydrolysis on ARF.

ARFGAP1 was the first ARF-GAP protein isolated [14]. This protein contains 415 amino acids, with a GAP catalytic domain located in the amino terminal 120 residues. ARFGAP1 was shown to localize primarily on Golgi membranes where it is thought to function in the formation of coated vesicles [15], [16]. The non-catalytic sequences of ARFGAP1 are necessary for associating with membranes. We have identified a region in the middle of the protein that is responsible for the membrane targeting of the whole molecule both *in vivo* and *in vitro* [17]. This region contains 132 residues starting from amino acid 203 to 334. Subsequently, a lipid-package sensor domain that allows the protein to sense membrane curvature was discovered in this region of the molecule [18].

Gcs1p is the ARF GAP in S. cerevisiae with the greatest sequence similarity to ARFGAP1 in its non-catalytic sequences. Besides its anticipated functions in the Golgi [19], [20], Gcs1p is also required for normal organization of the actin cytoskeleton [21]. *GCS1* interacts genetically with *SLA2* and *SAC6*. Mutants in the *SLA2* gene are defective in actin polarization and endocytosis [22], [23]. *SAC6* encodes an actin cross-linking protein, homologous to human fimbrin. In addition to this genetic evidence for a role for GCS1 in regulating actin, Gcs1p stimulates actin polymerization and inhibits depolymerization *in vitro* [21].

The Rho family of small GTPases, consisting of Cdc42, Rac and Rho, are the key regulators of the actin cytoskeleton [24]. Each of these small GTPases stimulates the formation of a unique type of actin morphology, although their activities are often coordinated. For example, Cdc42 stimulates the production of actin microspikes or filopodia [25]. Rac1 stimulates the formation of lamellipodia and membrane ruffles [26]. RhoA stimulates the formation of stress fibers and focal adhesions [27]. ARF6 has also been demonstrated to regulate the actin cytoskeleton. Earlier cell biology studies using GTP-binding mutants of ARF6 has demonstrated the important function that ARF6 regulates the reorganization of cortical actin [28], [29]. ARF6 appears to exert its effect on the cortical actin cytoskeleton by acting coordinately with Rac1 [30], [31]. ARF6 activates Rac by interacting with Rac GEFs or by regulating the availability of lipid rafts required for Rac attachment at the plasma membrane.

Of note, a subfamily of the ARF GAPs, called ARAP1, 2 and 3, have been shown to act as GAPs for both the ARF family small GTPases and the Rho family small GTPases Cdc42 and RhoA [4], [5]. ARAP1 is primarily localized on Golgi membranes, similar to ARFGAP1, suggesting that it might function in Golgi-derived vesicle trafficking. The presence of both an ARF GAP and a RhoGAP domain in ARAP proteins suggests that Class I ARFs and Rho proteins might interact functionally, as has been demonstrated for ARF6.

While studying the vesicle trafficking functions of ARFGAP1 we noticed that in cells overexpressing GAP273, a truncated protein containing only the carboxyl terminal 273 residues (residues 142–415) of ARFGAP1, cell spreading and flattening appeared to be impaired. Because the closest homologue of ARFGAP1 in yeast, Gcs1p, regulates the actin cytoskeleton as well as membrane traffic, we investigated the potential effect of ARFGAP1 on actin. We found that ARFGAP1, besides being localized on Golgi membranes, is present in the cell periphery associated with actin foci. We observed that cells over-expressing GFP-GAP273 are slow to spread when plated on fresh culture dishes. This effect is mediated by the carboxyl terminal 65 residues of ARFGAP1, and is related to the function of Rac1, as the over-expression of GAP273 inhibits the activation of Rac1.

## Results

### GFP-GAP273 inhibits cell spreading and is enriched in actin foci

**Figure 1:**
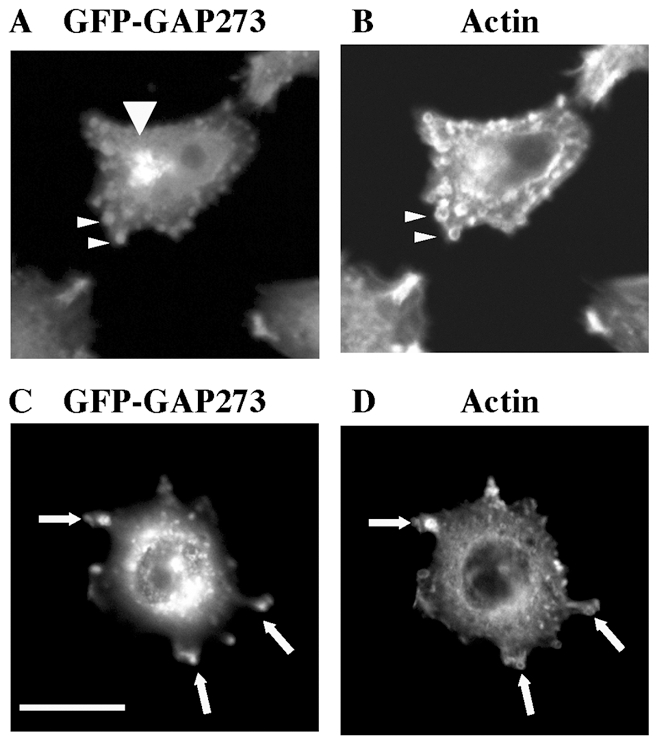
Co-localization of GFP-GAP273 with actin in the cell periphery. A, C. GFP fluorescence in CHO cells stably expressing GFP-GAP273 [GAP273(3.2)] cells. B, D. Actin staining of the same cells as in A and C, respectively. Scale bar  =  25 µm.

**Figure 2:**
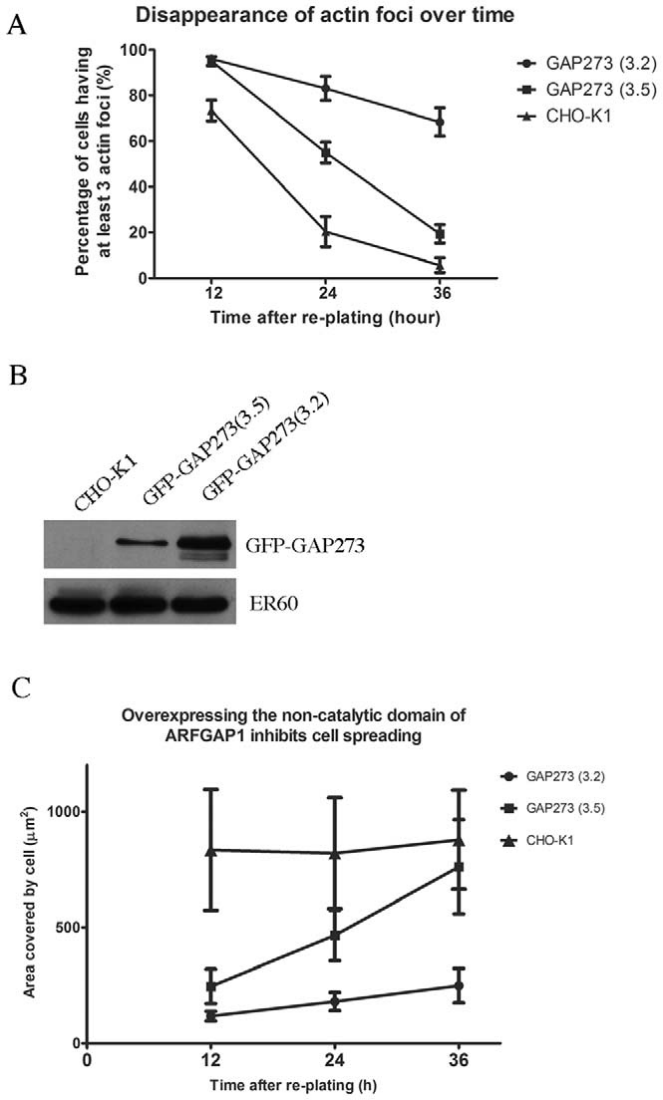
The ability of ARFGAP1 to stabilize the formation of cortical actin foci and inhibit cell spreading correlates with the level of expression of GFP-GAP273. A. Non-transfected CHO, 3.2 and 3.5 cells were plated into fresh culture dishes in medium containing serum. At the indicated intervals after plating, cells were fixed and stained with TRITC phalloidin. For each interval, more than 100 cells were observed and the fraction having 3 or more foci of peripheral actin is graphed as a function of time after plating. The data shown are from three independent experiments. Error bars  =  S.D. B. Relative expression levels of GFP-GAP273 in 3.2 cells and 3.5 cells was determined by immunoblotting of lysates from the indicated cells. GFP-GAP273 was detected by anti- GFP antibody. For a loading control, lysates were blotted with anti- ER60 antibody, which recognizes a 60 kDa ER resident protein. C. The mean surface areas occupied by CHO, 3.2 and 3.5 cells were measured at the indicated intervals after re-plating as described in Methods. A total of 50, 60, 60 cells from each time point for CHO-K1, 3.2, 3.5, respectively, were measured. Error bar  =  S.D.

We have previously generated Chinese Hamster Ovary (CHO) cell lines over-expressing a fusion protein consisting of the green fluorescent protein (GFP) at the amino terminus and a 273 amino acid, non-catalytic domain of ARFGAP1, called GAP273. In CHO cells stably transfected with GFP-GAP273, the fluorescent fusion protein was observed primarily on Golgi membranes in cells that had spread on their glass substrate [14], [17], [32]. However, we noticed that cells expressing GFP-GAP273 spread and flattened on their substrate slower than non-transfected CHO cells and often had a knobby appearance. These slowly spreading cells contained concentrated green fluorescence on both perinuclear membranes at the cis Golgi (Figure 1A, large arrowhead) and in peripheral foci near the plasma membrane (Figure 1A, small arrowheads) and at the tips of membrane protrusions (Figure 1C, arrows). The peripheral structures containing concentrations of GFP-GAP273 appeared to be surrounded by actin (Figure 1B and D). The presence of these actin-rich membrane “blebs”, which we will refer to as actin foci, decreased as a function of time after plating the cells and appeared to correlate inversely to cell spreading. As shown in Figure 2A, the percentage of cells having multiple (at least 3) actin foci decreased from about 73.3% (Standard deviation, S.D. = 4.5, n = 3) at 12 hours after re-plating to 5.7% (S.D. = 3.2%, n = 3) at 36 hours in wild type CHO cells (Figure 2A). In GFP-GAP273 cells, however, 96% (S.D. = 1.0%, n = 3) of the cells contained multiple actin foci at 12 hours after re-plating. 36 hours after re-plating this percentage decreased to about 68.3% (S.D. = 6.3%, n = 3) for GFP-GAP273(3.2) cells and 19.3% (S.D. = 4.0%, n = 3) for GFP-GAP273(3.5) cells (Figure 2A). In this experiment, at least one hundred cells were analyzed in each data point. 3.2 and 3.5 cells are two individually isolated clones from a transfection of GFP-GAP273 into CHO cells. Since the 3.2 cell line expressed a much higher level of GFP-GAP273 than the 3.5 cell line (Figure 2B), there was a positive correlation between the level of GFP-GAP273 expression and the frequency of cells having multiple actin foci. We further observed that the level of GFP-GAP273 expression inversely correlated with the ability of the GAP273 cells to spread after re-plating. In non-transfected CHO cells, the cells were efficiently spread out 12 h after re-plating. The mean area covered by the cells was approximately 800 µm^2^ (S.D. = 261 µm^2^, n = 50) at 12 h but did not increase over time (Figure 2C). The relatively large standard deviation is due to large variation in the size of the CHO cells. Two GFP-GAP273 clones showed delayed cell spreading with severities dependent on GFP-GAP273 protein expression. Clone 3.2 cells had a mean area covered by the cells of 117 µm^2^ (S.D. = 21 µm^2^, n = 60) at 12 h, and increased to approximately 249 µm^2^ (S.D. = 74 µm^2^, n = 60) at 36 h after re-plating. Clone 3.5 cells spread from 245 µm^2^ (12 h, n = 60) to 762 µm^2^ (36 h, n = 60). The observation of delayed cell spreading and numerous actin-rich membrane blebs caused by GFP-GAP273 was also confirmed when we overexpressed this construct in other cell lines such as Hela or HEK293 (data not shown).

**Figure 3:**
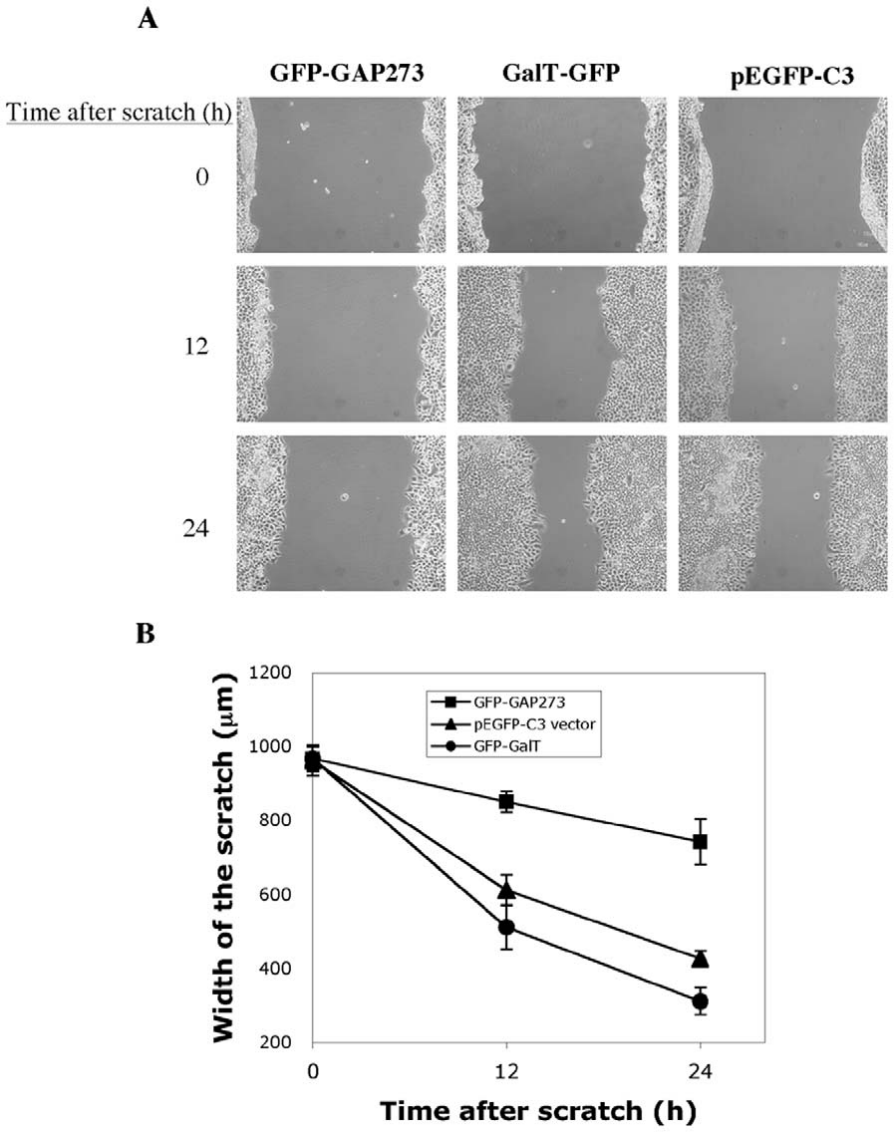
Cells over-expressing non-catalytic GFP-GAP273 migrate more slowly in an in vitro wound healing assay. Monolayers of HEK293 cells transiently transfected with GFP-GAP273, GFP-GalT, or pEGFP-C3 empty vector were scratched and the cell were allowed to spread into the scratched area. The degree of cell migration into scratched area was recorded at the indicated times. A. Images of cell migration over time. B. The average widths of the scratched area measured at various points are graphed. (n = 5, Error bars  = S.D.)

Cell migration requires extensive reorganization of actin. To determine if actin reorganization is compromised during migration in GFP-GAP273 cells, we conducted a cell migration experiment in which a monolayer of confluent cells was scratched and the migration of cells into the cleared area recorded as a function of time. As shown in figure 3, HEK293 cells transiently transfected with GFP-GAP273 are slow to close the scratched area (Figure 3A, left panels). Control cells that were transfected with GFP-GalT or GFP empty vector were able to migrate into and cover more than half of the scratched area in 24 hours (Figure 3A, middle and right panels, respectively). To quantify the extent of cell migration, we measured the gap of the scratched area at various points and then plotted the average width of the scratched area as a function of time after scratch. GFP-GAP273 clearly impeded cell migration in a statically significant manner (Figure 3B). These results further demonstrate that the reorganization of actin cytoskeleton during cell spreading and migration, is impaired by GAP273 overexpression.

To investigate the dynamics of membrane blebs caused by GFP-GAP273, we performed live cell imaging and recorded the appearance of such membrane blebs in cells transfected with GFP-GAP273 (movie S2). As a control, we also recorded images of cells transfected with GFP-Sec13, which has no known effect on actin (movie S1). As shown in the movie S1, GFP-Sec13 gives a largely diffused, cytosolic signal when overexpressed in actively spreading cells. Active membrane ruffling was present in the cell edges (boxed areas), suggesting that GFP-Sec13 overexpression does not inhibit this process. In contrast, in GFP-GAP273 overexpressing cells, the green fluorescent signal was concentrated in the perinuclear structures that likely represent the cis Golgi or the intermediate compartment, but diffused cytosolic signal was also observed. The extent of membrane movements was not reduced but the structures of membrane protrusions appeared to be much different from the GFP-Sec13 cells (movie S2). Spherically-shaped membrane protrusions containing green fluorescent signal and emerging at the cell edges were observed throughout the whole recording period (arrows). Active membrane ruffles similar to those found in GFP-sec13 cells were not obvious. These results confirm our initial observation that GAP273 must play a role in cell spreading and membrane ruffling, possibly by affecting the dynamics of actin reorganization at the cell cortex.

### Rac1 and ARF6 can suppress the effect of GAP273 on actin reorganization

**Figure 4:**
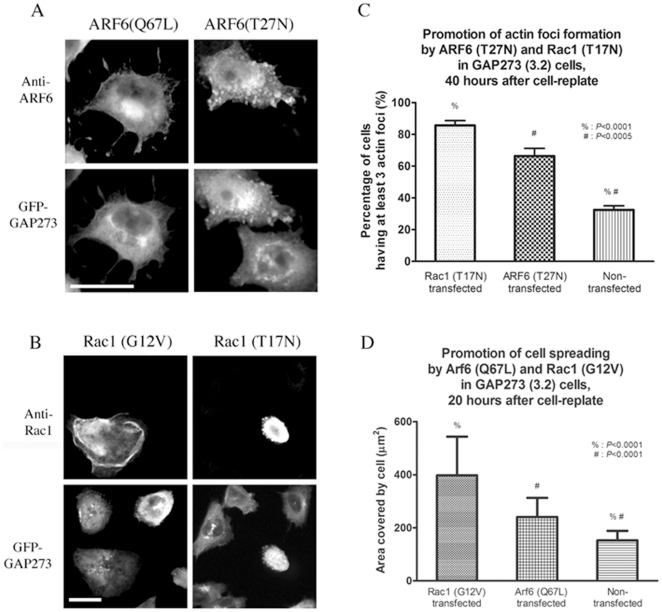
ARF6 and Rac1 can suppress the reorganization of the actin cytoskeleton by GAP273. A. Transient over-expression of ARF6(Q67L) in GFP-GAP273(3.2) promotes cell spreading (left panels). Actin foci are also absent from the cell. Transient over-expression of ARF6(T27N) in GFP-GAP273(3.2) cells (right panels) promotes or stabilizes the formation of actin foci. Images of GFP-GAP273 fluorescence of the same cells are shown in the bottom panels. The GFP-GAP273 fluorescence co-localizes with ARF6 staining in the cell transfected with ARF6(T27N). B. Transient over-expression of Rac1(G12V) (top left) promotes cell spreading and induces membrane ruffling in GFP-GAP273(3.2) cells but suppresses the localization of peripheral concentrations of GFP-GAP273 at the actin foci (compare the cell transfected with Rac1(G12V) with the surrounding non-transfected cells, bottom left). Transient over-expression of Rac1(T17N) (top right) in GFP-GAP273(3.2) cells inhibits cell spreading and prolongs the appearance of membrane projections containing GFP-GAP273. The GFP image (bottom right) shows that surrounding non-transfected cells are more spread and contain little, if any, peripheral concentrations of GFP-GAP273. For cells transfected with Rac1(G12V) or ARF6(Q67L), the cells had been re-plated on glass cover slips for about 20 hours before fixation and immunofluorescence labeling. For cells transfected with Rac1(T17N) or ARF6(T27N), the cells had been re-plated for 40 hours. Scale bar  =  25 µm. C. Quantification of the effect of Rac1(T17N) and ARF6(T27N) on the formation of actin foci. The percentage of cells transiently over-expressing Rac1(T17N) or ARF6(T27N) in GFP-GAP273(3.2) cells was measured. In each of the three independent experiments, one hundred cells were counted. Error bar  =  S.D. D. Quantification of the effect of Rac1(G12V) and ARF6(Q67L) on cell spreading. The area covered by GFP-GAP273(3.2) cells transiently over-expressed with Rac1(G12V) or ARF6(Q67L) was measured. The mean area covered were obtained from 41, 60 and 123 cells, for ARF6(Q67L), Rac1(G12V) or non-transfected GFP-GAP273(3.2), respectively. Error bar  =  S.D.

The actin cytoskeleton is regulated by members of the Rho family of small GTPases and by ARF6. Actin foci like the ones that we observed have been reported previously. They appeared in cultured cells that over-expressed a dominant negative, inactive allele of ARF6 (Supplementary Figure S1A) or Rac1 (Figure S1B and [4]). Moreover, a catalytically inactive mutant allele of ARF6 exchange factor ARNO, ARNO(E156K) [33], could also promote the appearance of actin foci when over-expressed (unpublished data). Over-expressing constitutively active alleles of ARF6 [ARF6(Q67L)] (Figure 4A, left panels) in GFP-GAP273(3.2) cells suppressed the formation of actin foci caused by GAP273 and stimulated cell spreading. In contrast, the inactive allele ARF6(T27N) promoted or perhaps stabilized the actin foci (Figure 4A, right panels). Similarly, the GFP-GAP273(3.2) cells over-expressing Rac1(G12V) were flat, extended and contained membrane ruffles, whereas the neighboring cells that were not transfected with the Rac mutant contained actin foci and were less spread out (Figure 4B, left panels). The inactive allele Rac1(T17N) promoted or stabilized the formation of actin foci just as did ARF6(T27N) (Figure 4B, right panels), and at the same time, inhibited cell spreading. We observed no change in cell spreading, the appearance of actin foci or the distribution of GFP-GAP273 in clone 3.2 cells transiently transfected with active or inactive alleles of ARF1 (unpublished data). The quantification of the results on cell spreading and actin foci formation shown in Figure 4A and 4B demonstrates statistically significant differences from data obtained from three independent experiments (Figure 4C and 4D). In GFP-GAP273 (3.2) cells, 33% (S.D. = 2.1%, n = 3) of the cells contain multiple (at least 3) actin foci 40 hours after re-plating. This percentage is much lower than that obtained at 36 hour after re-plating (compare 33% at 40 hours versus 68% at 36 hours, see Figure 2A). We think the difference may be an effect of adding fresh growth medium, which promotes cell spreading, to the cells after mock transfection. In cells transfected with either Rac1(T17N) or ARF6(T27N), 86% (S.D. = 3.0%, n = 3) and 66% (S.D. = 5.0%, n = 3) of the transfected cells have multiple actin foci, respectively (Figure 4C), suggesting that dominant negative mutants of Rac1 and ARF6 promoted or stabilized the actin foci, and concomitantly, inhibited cell spreading. The data shown here was collected from three independent experiments, in each of which at least 100 cells were analyzed for each data point. Cell spreading and membrane ruffling induced by activated alleles of ARF6 and Rac1 could suppress the formation of actin foci in GFP-GAP273 (3.2) cells. The areas occupied by ARF6(Q67L)- or Rac1(G12V)- transfected 3.2 cells were significantly larger than those occupied by the neighboring non-transfected cells within the same image (Figure 4D). On average, the Rac1(G12V)-transfected cells occupied 397 µm^2^ (S.D. = 146 µm^2^, n = 60), and ARF6(Q67L) occupied 241 µm^2^ (S.D. = 72 µm^2^, n = 41). However, the mean area covered per GFP-GAP273 (3.2) cell was 153 µm^2^ (S.D. = 36 µm^2^, n = 123). Therefore, constitutive Rac1 or ARF6 activity suppressed the inhibitory effect of GAP273 on cell spreading.

### ARFGAP1 functions upstream of Rac1

**Figure 5:**
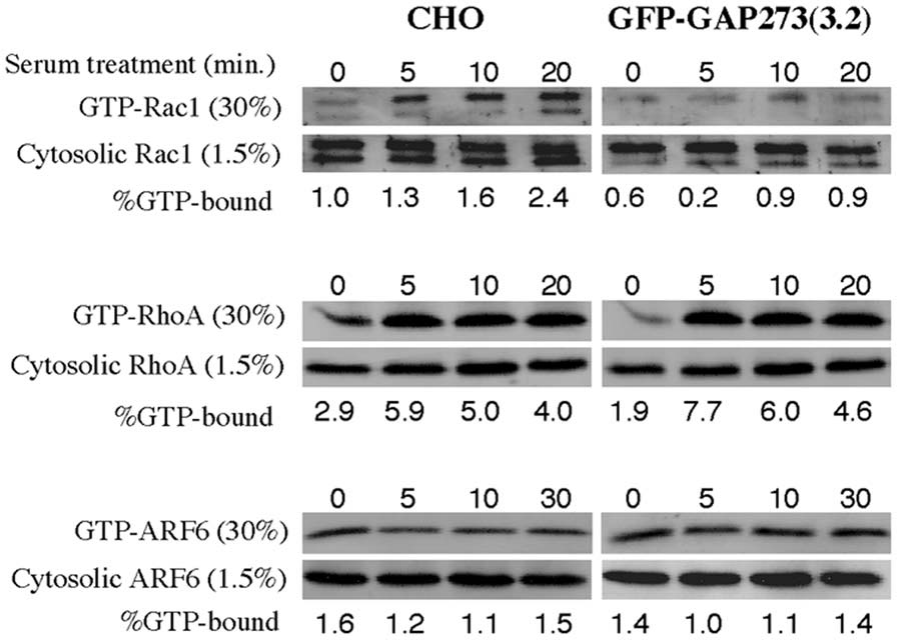
GAP273 inhibits the activation of Rac1 by serum. A. Activation of Rac1 is inhibited in CHO cells stably expressing GFP-GAP273, but not in wild type CHO cells. The result shown is a representative of three independent experiments. B. GFP-GAP273 did not inhibit the activation of RhoA. C. Serum caused little change in binding of GTP by ARF6 in either cell type and GFP-GAP273 did not affect ARF6 activation. The percent of total GTP-binding protein that was bound to GTP in each sample is listed below the image of the immunoblots.

The observation that the effect of GAP273 on the formation of actin foci could be suppressed by the constitutive activation of ARF6 or Rac1 might be explained by the hypothesis that ARFGAP1 might act as a negative regulator upstream of Rac1 and ARF6 in one or more signaling pathway(s). If so, one would expect a reduced fraction of GTP-bound Rac1 and/or Arf6 in GFP-GAP273 cells. To test this hypothesis, we compared the level of GTP-Rac1, GTP-ARF6, and GTP-RhoA *in vivo* in wild type CHO cells and in GFP-GAP273 (3.2) cells as a function of time after serum stimulation. If ARFGAP1 acted upstream of any of the small GTP-binding proteins tested, serum activation of that protein should be diminished in GFP-GAP273 (3.2) cells. In wild type CHO cells, Rac1 was activated by serum over the course of 20 minutes after serum was added to the cells. However, the serum activation of Rac1 was attenuated in the GFP-GAP273 (3.2) cells (Figure 5, top panels). Activation of another Rho family member, RhoA, by serum was not inhibited in GFP-GAP273 (3.2) cells compared to CHO cells (Figure 5, middle panels). Addition of serum did not increase the fraction of ARF6 bound to GTP in either cell line (Figure 5, bottom panels). Therefore, GAP273 affects actin cytoskeleton most likely via Rac1. The decreased activation of Rac1 in GFP-GAP273 (3.2) cells has been observed in three independent experiments. It is clear that the abnormal actin reorganization in GFP-GAP273 (3.2) cells is, at least in part, due to failure in Rac1 activation in these cells.

### The carboxyl-terminus of ARFGAP1 mediates the effect on actin

**Figure 6:**
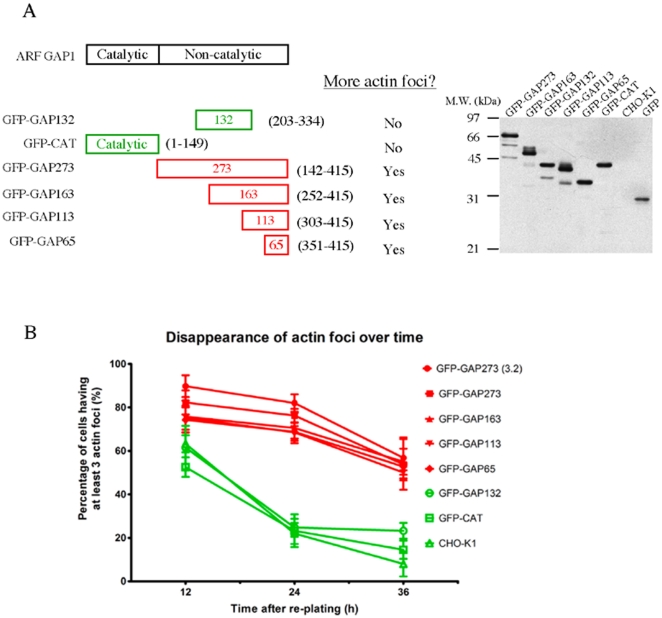
Various deletion mutants of ARFGAP1 were tested for their ability to prolong the appearance of cortical actin foci. A. Schematic diagram and information of various deletion mutants of ARFGAP1 tagged with GFP (left panel). Immunoblotting using anti-GFP antibody showed that the level of protein expression for these constructs was similar and no extensive degradation of the fusion proteins was observed. (right panel). B. Cells transiently transfected with the indicated GFP fusion constructs were analyzed for the presence of multiple (3 or more) actin foci after re-plating. The constructs having a stabilizing effect on actin foci are labeled in red to aid visualization. Those with no difference with wildtype CHO cells are labeled in green. 50–100 transfected cells were analyzed in each data point. GFP-GAP65 containing the carboxyl-terminal 65 amino acids of ARFGAP1 was able to increase the number of cells containing actin foci similar to GFP-GAP273. Three independent experiments were performed. Error bar  =  S.D.

**Figure 7:**
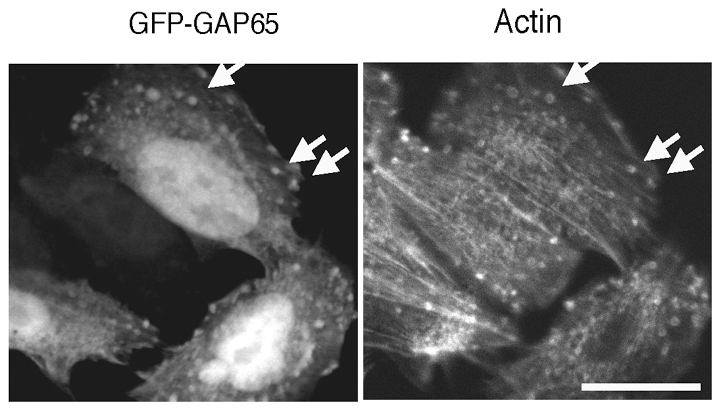
GFP-GAP65 co-localized with actin foci. GFP-GAP65 fluorescence is shown in the left panel and actin staining of the same cells is shown in the right panel, as indicated by the arrows. Scale bar  =  25 µm.

To determine the region of GAP273 responsible for the formation of actin foci, we measured the number of actin foci in cells transiently expressing various fragments of ARFGAP1 fused to GFP as a function of time after plating the cells (Figure 6). The information of this domain mapping experiment (left panel) and a control immunoblot showing the lack of extensive protein degradation of the various transfected ARFGAP1 fragment (right panel) is shown in Figure 6A. The carboxyl-terminal 65 amino acids of ARFGAP1 were sufficient to inhibit cell spreading and promote or prolong actin foci in CHO cells (Figure 6B). As controls, neither GFP-CAT, the amino-terminal one-third of ARFGAP1 that has GAP catalytic activity on ARF1, nor GFP-GAP132, the middle third that is sufficient for targeting to Golgi [17], stimulated the formation of actin foci. Three independent experiments were performed for each time points and transfected constructs. Typically 50 to 100 cells were analyzed in each of the independent experiments. Indeed, images of cells over-expressing GFP-GAP65 showed that GAP65 co-localized with actin foci (Figure 7, arrows). These results suggest that there are two separable functions in the non-catalytic domain of ARFGAP1, binding to Golgi membranes and interacting with the actin cytoskeleton.

## Discussion

In this report, we have demonstrated a novel effect of ARFGAP1 on the actin cytoskeleton. In cells transfected with the catalytically inactive, carboxyl terminal domain of ARFGAP1, GAP273, cell spreading was inhibited and more foci of cortical actin were observed in the transfected cells. Activation of Rac1 by serum was inhibited in cells over-expressing GAP273 and we saw no inhibition of RhoA or ARF6. The over-expression of constitutively active alleles of ARF6 or Rac1 suppressed the effect of GAP273 on actin. These observations are consistent with the hypothesis that ARFGAP1 affects actin cytoskeleton at least in part by inhibiting the activation of Rac1.

These results are surprising because the ARFGAP1 has never been postulated to function in the cell periphery involving in actin regulation. However, in view of the signaling capacity of ARF1 at the plasma membrane, it is possible that ARFGAP1, a molecule whose functions are intimately linked to ARF1, can regulate actin reorganization at the cell periphery. Other ARF GAPs have been extensively documented to regulate actin cytoskeleton. Among them, the best studied are GIT1, 2, and ASAP1. GIT1 and 2 bind to a number of molecules, through which they affect actin remodeling, cell spreading, focal adhesion turnover [34], [35], [36], [37]. ASAP1 has also been shown to affect these processes [38], [39], [40], [41].

Our results are consistent with the hypothesis proposed by others that small GTPases of the ARF and Rho families function coordinately *in vivo* both at the cell periphery and at the Golgi [4], [42]. Here, we have demonstrated that ARFGAP1 also acts in a bifunctional manner. It is a GAP protein for ARF1 and also inhibits the activation of Rac1, although at present we do not know how direct this effect may be. We do not believe that the effects of GAP273 on actin are due to an effect on ARF1. We observe no change in the kinetics of glycoprotein processing or transport in CHO cells stably over-expressing this protein, no change in the morphology of the Golgi in these cells. Transient expression of active or inactive alleles of ARF1 also does not change the actin phenotype of cells expressing GAP273 (unpublished data). Much higher over-expression of GAP273 is required to inhibit transport in the early secretory pathway [17]. Furthermore, ARFGAP1 has no detectable GAP activity towards ARF6 *in vitro* (unpublished data) and we observed no change in cellular pools of GTP-bound ARF6. If GAP273 were acting to inhibit GAP activity on ARF6, we would expect that the phenotype caused by over-expression of GAP273 would be mimicked by over expressing the constitutively active ARF6 allele, but we observed the opposite. Although it has been established that ARF6 functions to regulate cortical actin, our results show that the effect of ARFGAP1 is not directly mediated by ARF6. We speculate that ARFGAP1 may exert its effect on actin reorganization via an interaction with POR1/Arfaptin. POR1/Arfaptin was initially shown to interact with both Rac1-GDP and ARF6-GTP and regulate cytoskeleton rearrangement [43]. Subsequently it was shown to bind to ARF1, 5 and 6, but did not affect the GEF or GAP activities towards the ARFs [44].

There is a growing literature on the presence of, and requirements for, actin and actin-binding proteins in vesicles produced at the Golgi where ARFGAP1 is thought to function [42], [45], [46], [47], [48], [49]. ARF1 can directly [50] and indirectly [51] stimulate the production of phosphatidylinositol(4,5)bisphosphate on Golgi membranes, a lipid that is bound by several proteins that regulate actin polymerization as well as by proteins that regulate coated vesicle production. Thus, ARF1 has activities that could lead towards changes in the actin cytoskeleton. There is evidence that ARFGAP1 acts as an effector of ARF by interacting with vesicle cargo as well as coat proteins [52], [53]. Our observations suggest that an additional effector function of ARFGAP1 may be to link vesicle production to the local regulation of actin. An activated allele of Rac1 has been shown to inhibit membrane traffic to the apical, but not the basolateral surface in polarized epithelial cells [54]. This effect of Rac1 may not be limited to polarized epithelia, as fibroblasts maintain membrane traffic pathways analogous to those in polarized epithelia [55]. Another small GTPase of the same family, Cdc42, has been shown to regulate dyneine recruitment to COPI vesicles on Golgi [42]. However, the function of actin in coated vesicles is not completely understood. It has been noted previously that in addition to providing attachment sites for motors [56] or directly moving vesicles from the donor membrane through polymerization [57], [58], actin might also provide a barrier to vesicle formation [59]. Thus, whatever role actin plays in vesicle production, the local polymerization of actin and vesicle coats must be coordinated, and ARFGAP1 is a candidate for a component of this regulation.

Taken together, these results strongly indicate that the non-catalytic carboxyl terminus of ARFGAP1 is involved in regulating the function of actin cytoskeleton. This effect is likely mediated by inhibiting the activation of Rac1, a crucial regulator of actin cytoskeleton.

## Materials and Methods

### Cell cultures and transfections

Chinese hamster ovary CHO-K1 cells were grown in DMEM supplemented with 10 mM Hepes, pH 7.2, 0.35 mM L-proline, 5% fetal bovine serum (FBS), 50 U ml^−1^ penicillin and 50 U ml^−1^ streptomycin. Hela and HEK293 cells were grown in DMEM supplemented with 10% FBS. All three cell lines were obtained from the ATCC (Manassas, VA). Green fluorescent fusion proteins were made by inserting various fragments of ARFGAP1 cDNA into the expression plasmid pEGFP-C3 (Clontech). Transient transfections were carried out using 1 µg of plasmid DNA in a 6 well plate, together with transfection reagent Fugene 6 (Roche) or with polyethylenimine [60].

Plasmid pCMV3.1 containing cDNA of ARF6 (kindly provided by Julie Donaldson, NIH), myc-tagged-Rac1 mutant alleles (provided by Bill Singer and Paul Sternweis, U.T. Southwestern), or GFP fused in frame to various fragments of ARFGAP1 were transiently transfected into CHO cells. CHO cells stably expressing GFP-GAP273 have been previously described [17].

### Antibodies

Rabbit IgG against Rac1 was purchased from Millipore (Billerica, MA). Rabbit antiserum against ARF6 was obtained from Julie Donaldson (NIH). Mouse monoclonal antibody, 9E10, specific for a c-myc epitope was obtained from Yoav Henis (Tel Aviv University). Rabbit antiserum to ARFGAP1 was prepared as described [17] and IgG was isolated by affinity chromatography on GST-GAP273 protein. Rabbit anti-GFP antibody was purchased from Santa Cruz Biotechnology (Santa Cruz, CA). Rabbit antiserum against ER60 was obtained from Paul Kim (University of Cincinnati).

### Immunofluorescence and GFP imaging

Cells transfected with various GFP fusion proteins were grown on 11 mm diameter cover glasses. To detect GFP fusion proteins, cells were washed with PBS 3 times and then fixed in 3.7% formaldehyde for 15 minutes at room temperature. Formaldehyde was removed and the cells were incubated with serum free DMEM for 5 minutes at room temperature, rinsed in water and mounted on a glass slide.

For co-localization studies with actin, CHO cells expressing GFP-GAP273 were grown on cover glasses for the 16 h and then fixed in formaldehyde and permeabilized in 150 mM NaCl, 1 mM EDTA, 50 mM Tris HCl, pH 8.0, containing 1% bovine skin gelatin (NET/Gel) and 0.1% Triton X-100 for 10 minutes. For experiments involving the detection of actin, the samples were incubated with 0.5 ml of PBS plus TRITC-Phalloidin for 1 hour at room temperature.

In co-localization studies with ARF6 or Rac1, cells transiently transfected with DNA plasmids carrying cDNA for either protein were fixed and permeabilized as described and the samples were incubated with PBS plus 0.5% BSA for 15 minutes. Then rabbit polyclonal antiserum recognizing ARF6, or mouse monoclonal antibody 9E10 (to detect Myc-tagged Rac1) was applied to the samples. Co-localization with actin was achieved by adding TRITC-phalloidin in the incubation with primary antibody. Goat anti-rabbit IgG (for ARF6) and goat anti-mouse IgG (for Rac1) secondary antibodies (Invitrogen) conjugated with Alexa fluorophores were used to generate fluorescence signals. Images were collected with either a Biorad MRC 600 or a Carl Zeiss LSM5 confocal microscope at 630× magnifications, or an Olympus FV1000 at 600× magnifications. Double-labeled samples were excited with either a single 488 nm wavelength for recording images of GFP or Alexa 488 labels or with a 568 nm wavelength for imaging TRITC-phalloidin or Alexa 568 labels.

For real-time imaging of live cells expressing GFP-GAP273 or GFP-Sec13, 24 hours before transfection, 4×10^5^ Hela cells were seeded into each well of a 6-well plate. Transfection was done the next day with 3 µg DNA (GFP-GAP273 or GFP-Sec13) to 9 µg PEI in a total volume of 200 µl PBS. The transfected cells were then trypsinized 6 hours later and plated to a 35 mm petridish, having a 14 mm microwell attached with a 0.16–0.19 mm thick coverslip (MatTek). After 16 hours of incubation, GFP signals from the transfected cells was monitored by real time confocal imaging technique from an Olympus FV1000 confocal microscope system at 600× magnifications. Images were taken in a 30 seconds interval for a total period of 15 minutes.

### Measuring actin foci and cell spreading

The experiments shown in Figures 2 examined CHO cell lines stably expressing different levels of GFP-GAP273 [17]. The experiments shown in Figure 6 examined the effects of transient expression of truncated GFP-GAP proteins in CHO cells. To measure actin foci, for both sets of experiments CHO cells were removed from 6-well plastic culture dishes and approximately 2×10^4^ cells were re-plated onto 11 mm cover glasses. At the indicated intervals after plating, the cells were fixed and stained with TRITC-phalloidin to visualize actin. Cells with three or more actin foci were counted and expressed as a percentage of the total number of cells analyzed. In every case, more than one hundred cells were analyzed for each time point. Three independent experiments were performed to obtain statistical data.

Cell spreading was measured by the area occupied by individual cells. The area of a cell was traced with the outline of the cell image using NIH Image J v1.43. The area under this outline was quantified and converted from pixels to area unit (µm^2^) based on the magnification and the aid of scale bar.

### *In vitro* wound healing assay

24 hours before transfection, 8×10^5^ HEK293 cells were seeded into each well of a 6-well plate and incubated in 5% CO_2_ incubator at 37°C overnight. Before further experiment, each well was examined to ensure that a monolayer of cells was completely covering the well surface. Transfection was done with 3 µg DNA (GFP-GAP273) to 9 µg PEI in a total volume of 200 µl PBS. A scratch was then made across the cell monolayer with a 10 µl pipette tip and the medium was changed to remove any cell debris left behind. Brightfield images of the scratch were taken after 0, 12 and 24 hours, respectively.

### Preparation of the Rac-binding GST-PBD reagent

*E. coli* carrying the plasmid pGEX-PBD, which encodes GST fused to the CRIB domain of p65PAK, that binds to Rac and CDC42 proteins [61], were grown to OD_600_ between 0.6–0.8 at 37°C in 200 ml of Luria broth containing 50 µg/ml carbenicillin. Protein expression of GST-PBD was induced with 0.3 mM of IPTG for 3 hours at 37°C. The bacteria were harvested by centrifugation and resuspended in lysis buffer containing 20 mM HEPES, pH 7.5, 120 mM NaCl, 10% glycerol, 2 mM EDTA, 1 mM PMSF, and protease inhibitor cocktail Complete® (Roche Diagnostics, IN) at 4°C. The bacterial cells were broken open by sonication. Cell debris was pelleted at 30,000 g for 30 minutes at 4°C. The supernatant was taken and NP-40 was added to 0.5% of the final volume. 0.3 ml bed volume of glutathione-sepharose beads was added to the supernatant and the mixture was incubated at 4°C with gentle agitation for 1 hour. Then, the beads were washed with PBD lysis buffer plus 0.5% NP-40 for five times and lysis buffer (without NP-40) for 3 more times. The purified GST-PBD bound to glutathione beads could be frozen as a 50% slurry in PBD lysis buffer at −80°C.

### Preparation of the Rho-binding GST-RBD reagent

A 100 ml culture of bacteria carrying the plasmid pGEX-RBD was grown to OD 0.6–1.0 at 37°C, and GST-RBD protein [62] expression was induced by 0.3 mM IPTG at room temperature for 3 hours. The bacteria were harvested by centrifugation and resuspended in RBD lysis buffer containing 50 mM Tris, pH 7.4, 50 mM NaCl, 5 mM MgCl_2_, 1 mM DTT, 1% Triton X-100, and protease inhibitor cocktail Complete® at 4°C. The bacterial cells were broken open by sonication. Cell debris was pelleted at 22,000 g for 30 minutes at 4°C. The supernatant was incubated with 0.5 ml bed volume of glutathione-sepharose beads for 1 hour at 4°C. Then, the beads were washed with RBD lysis buffer three times and with lysis buffer without Triton X-100 for an additional three times. The purified GST-RBD bound to glutathione beads could be frozen as a 50% slurry in RBD lysis buffer (without Triton X-100) at −80°C.

### Preparation of the ARF-binding GST-GGA3_VHS-GAT_ reagent

GST-GGA3_VHS-GAT_ was prepared from bacteria carrying the plasmid pGEX-GGA3_VHS-GAT_, using procedure described in Puertollano et al. [63].

### Determination of the fraction of GTP-bound small GTPase *in vivo*

CHO K1 cells or GFP-GAP273 cells at 70% confluency in a 100 mm plate were starved with serum overnight in DMEM supplemented with 10 mM Hepes, pH 7.2, 35 mM L-proline, 50 U ml^−1^ penicillin and 50 U ml^−1^ streptomycin. The next day the cells were treated with the same medium supplemented with 15% fetal bovine serum for intervals between 0 and 30 min at 37°C. The cells were then placed on ice, quickly washed with PBS once and lysed with 0.55 ml of ice-cold buffer A (50 mM Tris, pH 7.6, 500 mM NaCl, 0.1% SDS, 0.5% deoxycholate, 1% Triton X-100, 10 mM MgCl_2_, and protease inhibitor cocktail Complete®). The lysates were scraped from the plates and collected into microfuge tubes. Insoluble material was removed by centrifugation at 24,000 g for 10 min at 4°C. 0.5 ml of the supernatants was transferred to another microfuge tube containing 40–50 µg of glutathione beads loaded with GST-PBD (for the isolation of Rac1-GTP), GST-RBD (for RhoA-GTP), or GST-GGA3_VHS-GAT_ (for ARF6-GTP or ARF1-GTP). The mixtures were incubated at 4°C for 30 minutes with gentle agitation. The remaining 50 µl of supernatants were mixed with equal volume of 2X SDS sample buffer and would be used to measure the total amount of Rac1, RhoA or ARF6 present in the lysate. After the incubation, the beads were washed with ice-cold buffer B (50 mM Tris, pH 7.6, 150 mM NaCl, 1% Triton X-100, 10 mM MgCl_2_, and protease inhibitor Complete®) four times. After the last wash, 30 µl of 2X SDS sample buffer was added to the beads, which typically had a volume of 15 µl. Finally, the samples were subjected to SDS-PAGE and immunoblot analysis using enhanced chemiluminescence. Films were scanned with a Molecular Dynamics laser scanning densitometer or a Bio-Rad GS-800 densitometer and quantified with ImageQuant software (Molecular Dynamics).

## Supporting Information

Figure S1 Actin foci when ARF6 or Rac1 are inactivated. A. Over-expression of ARF6(Q67L) (top left) promotes cell spreading in CHO cells and eliminates actin foci. Over-expression of ARF6(T27N) (top right and bottom panels) induces the formation of numerous actin foci. ARF6(T27N) (bottom left) co-localizes with actin foci (bottom right). B. Over-expression of Rac1(G12V) (top left) promotes cell spreading and membrane ruffling. Over-expression of Rac1(T17N) (bottom left) promotes the formation of and co-localizes with actin foci (bottom right). The expression of ARF6 and Rac1 was detected by rabbit antiserum specific against ARF6 and mouse monoclonal antibody (9E10) against the myc-tag on Rac1. Antibodies were detected with secondary antibodies conjugated with Alexa 488. Actin staining was detected by TRITC-conjugated phalloidin. Scale bar  =  25 µm.
(TIF)

Movie S1 HEK293 cells overexpressing GFP-Sec13. Images were recorded at 30-second intervals for 15 minutes. Membrane ruffling activity is very obvious throughout the recording period (boxed areas). There are no observable spherical membrane structures at the cell periphery.
(AVI)

Movie S2 HEK293 cells overexpressing GFP-GAP273. Images were recorded at 30-second intervals for 15 minutes. Spherical membrane structures containing green fluorescence signal were rapidly appearing and retracting at the cell periphery (arrows). These protrusions seldom generated actin microspikes. Active membrane ruffling was not obvious in these cells.
(AVI)
