# Supplementary figures and images for: The Non-Catalytic Carboxyl-Terminal Domain of ARFGAP1 Regulates Actin Cytoskeleton Reorganization by Antagonizing the Activation of Rac1
Source: PLoS One. 2011 Apr 4;6(4):e18458. doi: 10.1371/journal.pone.0018458 (PMC3070737; doi:10.1371/journal.pone.0018458)

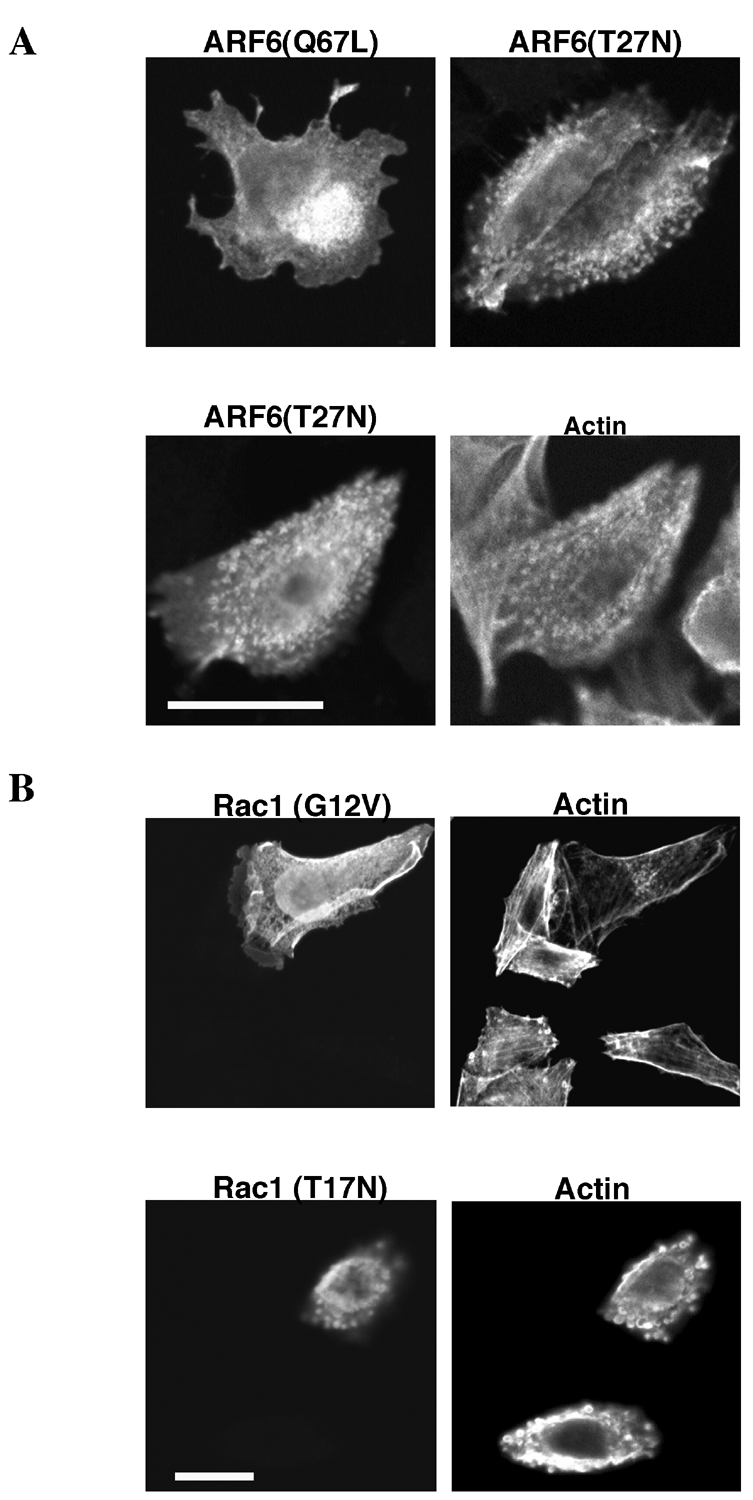

Supplement: Figure S1 — Actin foci when ARF6 or Rac1 are inactivated. A. Over-expression of ARF6(Q67L) (top left) promotes cell spreading in CHO cells and eliminates actin foci. Over-expression of ARF6(T27N) (top right and bottom panels) induces the formation of numerous actin foci. ARF6(T27N) (bottom left) co-localizes with actin foci (bottom right). B. Over-expression of Rac1(G12V) (top left) promotes cell spreading and membrane ruffling. Over-expression of Rac1(T17N) (bottom left) promotes the formation of and co-localizes with actin foci (bottom right). The expression of ARF6 and Rac1 was detected by rabbit antiserum specific against ARF6 and mouse monoclonal antibody (9E10) against the myc-tag on Rac1. Antibodies were detected with secondary antibodies conjugated with Alexa 488. Actin staining was detected by TRITC-conjugated phalloidin. Scale bar = 25 µm. (TIF) [file pone.0018458.s001.tif]
